# Finite element simulations of smart fracture plates capable of cyclic shortening and lengthening: which stroke for which fracture?

**DOI:** 10.3389/fbioe.2024.1420047

**Published:** 2024-07-23

**Authors:** Michael Roland, Stefan Diebels, Kerstin Wickert, Tim Pohlemann, Bergita Ganse

**Affiliations:** ^1^ Chair of Applied Mechanics, Saarland University, Saarbrücken, Germany; ^2^ Department of Trauma, Hand and Reconstructive Surgery, Departments and Institutes of Surgery, Saarland University, Homburg, Germany; ^3^ Werner Siemens-Endowed Chair for Innovative Implant Development (Fracture Healing), Departments and Institutes of Surgery, Saarland University, Homburg, Germany

**Keywords:** fracture healing, bone regeneration, computer simulation, smart implant, active implant, biomechanics, osteosynthesis, digital health

## Abstract

**Introduction:** Bone healing can be improved by axial micromovement, as has been shown in animals and human patients with external fixators. In the development of smart fracture plates, the ideal amount of stroke for different fracture types in the different healing stages is currently unknown. It was hypothesized that the resulting strain in the fracture gap of a simple tibial shaft fracture does not vary with the amount of axial stroke in the plate, the fracture gap size, and the fracture angle.

**Methods:** With finite element simulations based on body donation computed tomography data, the second invariant of the deviatoric strain tensor (J2), strain energy density, hydrostatic strain, octahedral shear strain, and percentage of the fracture gap in the “perfect healing window” were computed for different gap sizes (1–3 mm), angles (5°–60°), and plate stroke levels (0.05–0.60 mm) in three healing stages. Multiple linear regression analyses were performed.

**Results:** Findings showed that an active fracture plate should deliver an axial stroke in the range of 0.10–0.45 mm. Different optimal stroke values were found for each healing phase, namely, 0.10–0.25 mm for the first, 0.10 mm for the second, and 0.35–0.45 mm for the third healing phase, depending on the fracture gap size and less on the fracture angle. J2, hydrostatic strain, octahedral shear strain and the strain energy density correlated with the fracture gap size and angle (all *p* < 0.001). The influence of the fracture gap size and angle on the variability (adjusted R^2^) in several outcome measures in the fracture gap was shown to vary throughout healing. The contribution to the variability of the percentage of the fracture gap in the perfect healing window was greatest during the second healing phase. For J2, strain energy density, hydrostatic strain, and octahedral shear strain, the fracture gap size showed the greatest contribution in the third fracture healing phase, while the influence of fracture angle was independent of the healing phase.

**Discussion:** The present findings are relevant for implant development and to design clinical studies that aim to accelerate fracture healing using axial micromovement.

## 1 Introduction

Fracture healing is a physiological process that takes place in stages and involves numerous cellular components (Saul et al., 2023). Healing phases are the acute inflammatory response, recruitment of mesenchymal stem cells, generation of a cartilaginous and a periosteal bony callus, revascularization and neo angiogenesis at the fracture site, and mineralization and resorption of the cartilaginous callus, followed by bone remodeling (Marsell and Einhorn, 2011). The healing process usually requires several weeks to months, and in around 5%–10% of cases, fractures do not heal with the result of a non-union (Zura et al., 2016). Such healing problems are associated with massive socio-economic costs (Rupp et al., 2018; Leliveld et al., 2020). Factors associated with an elevated risk for non-union include open and more severe fractures, a high body mass index, smoking, and alcoholism (Zura et al., 2016). Excessive movement and forces in the fracture may also lead to non-union (Claes et al., 1998; Foster et al., 2021). To decrease costs, to bring patients back to work faster and to shorten immobilization time, it is desirable to reduce the incidence of non-union, as well as to shorten the healing time of bone fractures. In human tibial fractures, daily application of controlled cyclic micro-movement with an axial stroke of 0.5 mm via external fixators reduced the healing time by 21%–23% (Kenwright et al., 1986; Kershaw et al., 1993). In detail, Kenwright et al. (Kenwright et al., 1986) found faster healing by 7.1 weeks (23%, 30.8 weeks healing time without and 23.7 weeks with stimulation, 50 and 32 patients, respectively) and Kershaw et al. (Kershaw et al., 1993) reported a facilitation by 6 weeks (21%, 29 weeks healing time without and 23 weeks with stimulation, 23 and 22 patients, respectively). In addition, a decrease in the incidence of non-union was reported (8 non-union cases without and 2 cases with micro-movement) (Kenwright et al., 1986).

It is known that axial compression is better for fracture stimulation than translational shear or distraction (Augat et al., 2003; Hente et al., 2004; Bishop et al., 2006; Sigurdsen et al., 2011). According to findings from animal and human studies, the ideal axial stroke in the fracture gap seems to be around 0.4–0.5 mm, depending on the fracture gap size (Kenwright and Goodship, 1989; Wolf et al., 1998). However, this has not yet been systematically studied *in silico* for different fracture types and healing phases. Changes in the ideal stroke value of an active plate over time have, to the authors’ knowledge also never been determined for human bones. Increasing fracture gap sizes are known to delay the healing process (Claes et al., 1998). In addition, the angle of obliquity θ) of a fracture has significant effects on interfragmentary movement (IFM) and on octahedral shear strain (Miramini et al., 2016). Thus, the fracture gap size and the angle of obliquity might need to be considered when planning active fracture stimulation with axial micromovement by an implant, and when determining the stroke, the implant needs to deliver. Claes & Heigele (Claes and Heigele, 1999) suggested that intramembranous bone formation occurs when strains smaller than ±5% and hydrostatic pressures smaller than ±0.15 MPa are present in the local tissue. In addition, endochondral ossification was suggested to be associated with compressive pressures larger than ±0.15 MPa and strains smaller than ±15%. Claes & Heigele proposed that all other conditions lead to the formation of connective tissue or fibrous cartilage instead of bone. Their idea of a so-called ‘perfect healing window’ was based on animal experiments, cell culture studies, and finite element models (Claes et al., 1998). The underlying mechanobiological mechanisms in bone regeneration include stimulation of mechano-sensitive Piezo channels that are involved in activating pathways in osteoblasts under cyclic stretching (Kang et al., 2024). Numerous cellular components, growth fractures and cytokines induce vascularization of the initially hypoxic fracture callus within 2–5 weeks, supporting ossification (Menger et al., 2022a). When forces and movement in the fracture gap are too high, the newly formed bony structures may be destroyed and need to be re-built, which is why excessive strains lead to the formation of connective tissue.

External fixators, as used in the named micro-movement studies, are frames that stabilize the fracture outside the skin and that are attached to screws and/or wires that connect them to the bone (Fernando et al., 2021). Among their disadvantages are a low patient comfort due to the external frame, risks of pin-site infections, and implant breakage or loosening. Ideally, it would be possible to provide the same stimuli through internal implants, similar to the nails and plates currently used as the gold standard in surgical fracture treatment. Furthermore, it would be desirable, if the implant had sensor functions, e.g., for stiffness changes and daily movement time in the fracture gap, and if it could process the data and act autonomously according to the individual current biomechanical needs (Ledet et al., 2018; Windolf et al., 2022). Smart implants with sensing and acting capabilities are currently under development to allow live monitoring of the fracture healing progress, as well as direct mechanical interventions (Ernst et al., 2021; Ganse et al., 2022). Technological advances in material science and systems engineering have recently opened up new opportunities to build smart fracture implants (Ganse et al., 2022). For example, shape memory alloys (SMA), such as Nitinol have the ability to shorten if warmed up, e.g., by an electric current, and it is possible to measure changes in their electrical resistance that correlate with the changes in length (Motzki et al., 2018). SMA wires embedded in a fracture plate or nail would allow the implant to shorten and to change its stiffness. This technology could not only be used to stimulate bone healing at the fracture site by applying cyclic load, but it could also be applied to vary the implant stiffness during the course of healing, e.g., by moving stiffer and less stiff elements within the implant or by activating SMA wires embedded in composite materials. The right changes in implant stiffness during the healing process are known to enhance healing (Barcik and Epari, 2021; Fu et al., 2023).

As the authors are currently working on the development of an active fracture plate capable of shortening and lengthening, they needed to know which amount of axial stroke the mechanism needs to be able to deliver (Ganse et al., 2022). To be able to generate the optimal resulting cyclic movements in the fracture gap by an active plate, depending on fracture geometry, more or less stroke might be required in the plate. Currently, the amount of plate shortening needed for different fracture types and orientations is unknown. The authors are not aware of any previous papers that determined effects of an active fracture plate on the biomechanical environment in different fracture types. Therefore, the null-hypothesis of the present study was that the resulting strain in the fracture gap of a simple tibial shaft fracture does not vary with the amount of axial stroke in the plate, the fracture gap size, and the fracture angle.

## 2 Materials and methods

Ethical approval was obtained from the IRB of Saarland Medical Board (Aerztekammer des Saarlandes, Germany, application number 146/21). The study is part of the project Smart Implants, funded by the Werner Siemens Foundation. It is registered in the German Clinical Trials Register (DRKS-ID: DRKS00025108).

### 2.1 Model generation

As strain inside a fracture cannot be measured in experiments, it was decided to work with finite-element simulations. The authors have improved and validated their finite element simulations with numerous test stand experiments over the past years (Braun et al., 2021; Orth et al., 2023; Roland et al., 2023; Wickert et al., 2024). The workflow for model generation was divided into four main steps: 1) the bone model generation based on computed tomography data, 2) the design of a virtual twin of an active implant, 3) the generation of different fractures and 4) the assignment of material parameters representing different healing phases.

#### 2.1.1 Bone model

**FIGURE 1:**
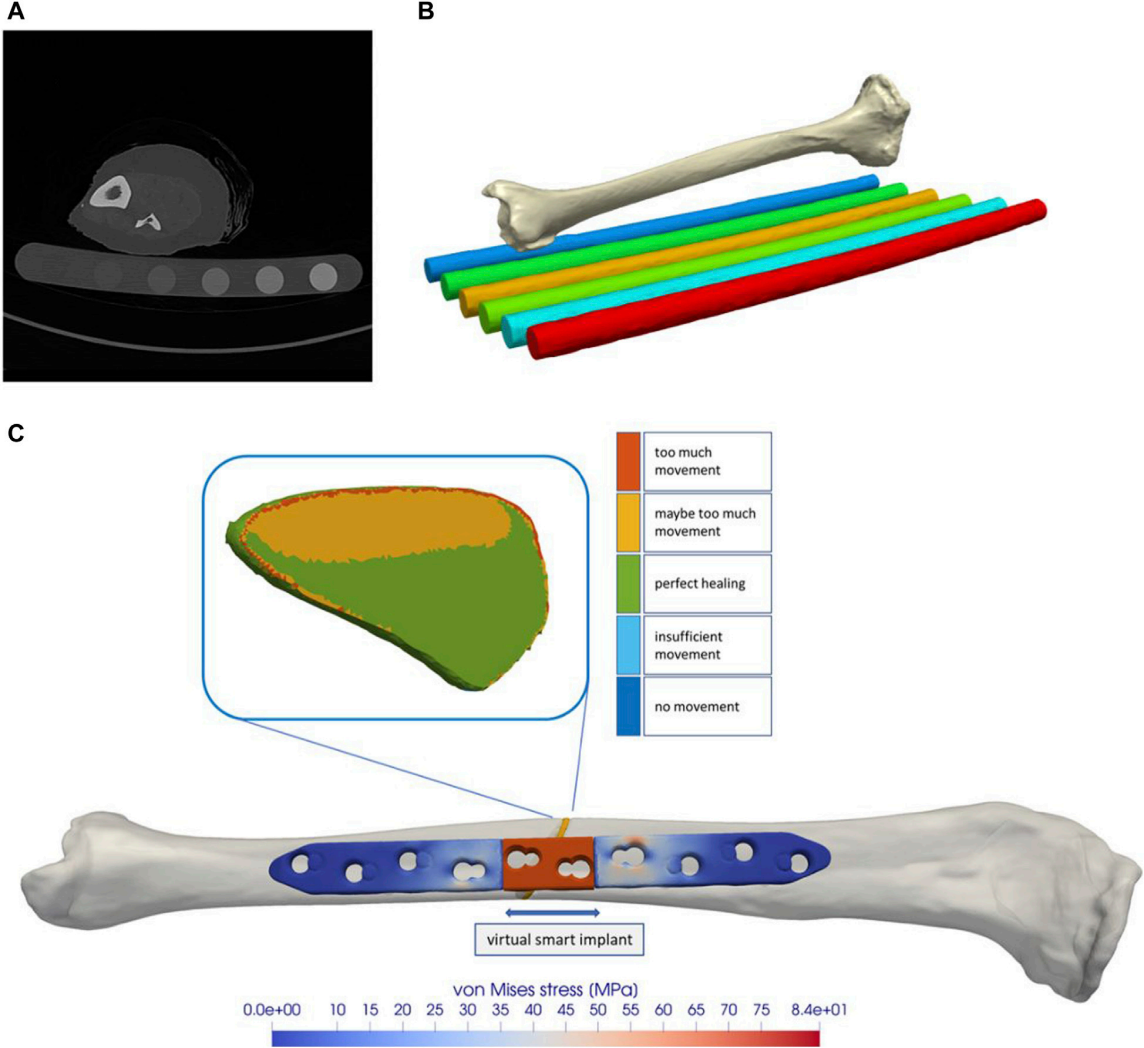
The tibial model was generated by processing computed tomography data that were recorded with a phantom for bone density estimation **(A,B)**. The model of a regular state-of-the-art bone plate was adapted to simulate shortening of the part depicted in red **(C)**. The fracture gap and the healing-window classification are shown as an example.

To obtain a realistic bone model, a computed tomography (CT) scan (SOMATOM Definition Edge, Siemens, Erlangen, Germany) of a human cadaveric specimen combined with a six-rod bone density calibration phantom (QRM-BDC/6, QRM GmbH Moehrendorf, Germany) was used. The donor was a female at the age of 74 years with a body height of 152.4 cm (60 in.) and a body weight of 81.65 kg (180 lbs.) without any known bone disease that could have had a lasting negative effect on bone quality. The CT scan was performed with a resolution of 0.541 mm of pixel spacing and 0.60 mm distance between two images, cf. Figure 1 A. The image stack was segmented with an adaptive threshold procedure with respect to the calibration phantom, supplemented by a morphological close filter with isotropic values and a mask smoothing with a recursive Gaussian filter with anisotropic values. Afterwards, an island-removal, a cavity-fill and a fill-gaps filter with priority order procedure were applied, resulting in a high segmentation quality without detectable problems, cf. Figure 1 B. All image processing steps were performed in the software ScanIP (Synopsys, Mountain View, CA, United States).

In order to provide suitable material parameters in the finite element (FE) models, up to four steps are necessary: the first step is a densitometric relationship defining a mapping to convert raw CT attenuation to bone mineral density (BMD) values with the help of the six-rod calibration phantom. For this purpose, histograms were generated for the individual rods, representing the corresponding grayscale values from the raw CT data, given in Hounsfield units (HU), with respect to their number of voxels. The histogram curves were then smoothed with a robust local regression method in Matlab (Matlab 2021b; MathWorks, USA). Then, the maxima of each regression curve were used as calibration points for a least square fit defining a linear mapping of the CT data in HU to equivalent mineral density values (ϱ_eqm) also often referred to as quantitative CT values. The result of this calibration process is given by the linear mapping:

$$\varrho \_\text{eqm}=a\cdot \text{HU}+b,\text{with }a=7.939e‐04\text{ and }b=\left.0.014\right.$$

The second step calculates the associated ash density (ϱ_ash) values from the calibrated equivalent mineral density values. For this purpose, the following relationship for hydroxyapatite phantoms was implemented (Eberle et al., 2013):

$$\varrho \_\text{ash}=0.8772\cdot \varrho \_\text{eqm}+\left(0.0789\right)$$

The third step is the conversion of the ash density into the apparent density (ϱ_app) which is used in the most relationships computing material parameters. Here, the conversion from (Edwards et al., 2013), originally defined by (Dalstra et al., 1993) was used:

$$\varrho \_\text{app}=\varrho \_\text{ash}/\left.0.626\right.$$

The last step is the mapping from the apparent density (ϱ_app) to the Youngs modulus and Poisson ratio. Since most published formulas are only defined for the density range of the cancellous bone (Eberle et al., 2013), the formula of Rho et al. (Rho et al., 1995),

E=6,570⋅ϱ_app^ 1.37

with a Poisson ratio of 0.30 was used here. In accordance with (Cattaneo et al., 2001), for the bone 25 different material cards were generated via the described workflow and stored in the FEM model.

#### 2.1.2 Virtual twin of an active implant

Since there is currently no approved implant with active components on the market, a virtual implant of this kind was generated here. This enables the effects of such an implant on fracture healing to be tested by means of simulations and thus to gather initial experience for a later design of the components. To implement this concept, a CAD model of a standard implant (LCP locking compression plate) was created and an active element was added in the middle part. Afterwards, the virtual implant in the simulated environment was placed on the bone model by an orthopaedic trauma surgeon analogous to a real application in the software ScanIP using the CAD import module. Six screws, three on each side of the fracture, were additionally integrated into the model, cf. Figure 1 C. The virtual active element is realized in the simulation process by a suitable choice of boundary conditions. Therefore, the predefined stroke is set via Dirichlet boundary conditions on the distal side of the active element and complemented with fixed Dirichlet boundary conditions on the proximal side. Since this work focuses on the strain state of the fracture, we omitted the definition of contact conditions and rigidly connected the individual masks in the simulation. For the simulations, it was assumed that the active implant is made from the standard material Ti6Al4V, with a Young’s modulus of 108,000 MPa and a Poisson’s ratio of 0.37.

#### 2.1.3 Generation of different fractures

To investigate the influence of an active implant on fracture healing, different fractures were virtually generated and also placed by an orthopaedic trauma surgeon. For this purpose, a STL (stereolithography or standard triangle language) file of the bone surface was generated from the segmented bone model and processed in a free-from software (Geomagic Freefrom Plus/Touch X, 3D Systems, Inc.). The freeform software is oriented on clay modelling and offers various analogue tools for editing the 3D objects. Therefore, clay-based modelling was chosen here and the 3D scraper tool was used to create the fractures in the 3D clay object based on the STL file. The generated fractures were then saved again as independent STL files. They were then loaded into the ScanIP software to the segmented bone models and the CAD-based active implant. Using a surface-to-mask operation on the individual fractures and a subsequent Boolean operation between the masks for bone and fracture, the corresponding 3D models were created for each fracture.

#### 2.1.4 Mapping of different healing phases

The FEM simulations were conducted as three separate statical simulations and not as a longitudinal process. Three healing states were mapped virtually, as they may require different strokes of the active implant as a suitable mechanical stimulus. Starting from initial connective tissue with the material parameters Youngs modulus E = 3.0 MPa and Poisson ratio of 0.4 (Claes et al., 1998), the next class of fracture gap material considered here is fibrous cartilage with a Youngs modulus of E = 200 MPa and a Poisson ratio of 0.45 (Simon et al., 2011), and the last considered material is soft callus with Youngs modulus of E = 1,000.0 MPa and Poisson ratio of 0.3 (Claes et al., 1998).

#### 2.1.5 Generation of the 3D FE meshes

Meshing of the 3D models was also performed in the ScanIP software. For this purpose, the segmented masks were placed in priority order, in ascending sequence from bone to fracture to implant. To increase the resolution of the fracture, an adaptive refinement was selected in all generated models, with the volume meshing parameter ‘Coarseness’ of −10 for bone and implant and −5 for the fracture area. This resulted in grids with around 1,000,000 mesh cells, whereby the number of mesh cells varied depending on thickness and angle of the fracture mask. Since all simulations were carried out in the simulation environment Abaqus (Dassault Systèmes, Velizy-Villacoublay, France), all meshes were generated using quadratic finite elements (C3D10, ten-node tetrahedral element with four integration points) with respect to the adaptive mesh resolution. At the beginning of the meshing process, two regions of interest (ROI) were marked on the active element of the implant and saved as node sets in the Abaqus input files to be able to apply the boundary conditions in the correct way. For every mesh, the software ScanIP checked the mesh statistic and the segmented masks for errors and all volume mesh generations were successful without detectable problems.

#### 2.1.6 Biomechanical simulations

All simulations were performed in Abaqus using a standard workstation computer (Intel(R) Core(TM) i9-9920X CPU @ 3.50GHz, 128GB RAM, 64-Bit Windows 10 Pro) in sequential order in a queue driven by a Python script. The post-processing of the results, stored in Abaqus OdB (output database) files, was realized using Python in-house software. The relevant strain variables, which describe the mechanical stimulus or the interfragmentary movement, were read out for each integration point of the mesh cells of the fracture area and processed for statistical evaluation.

### 2.2 Statistics

The weighted means of the outcome measures described in Table 1 were calculated in the simulations. The perfect healing window was computed according to the papers of Claes & Heigele (Claes and Heigele, 1999) and Claes et al. (Claes et al., 1998). In the tibial model, transverse fractures with different gap sizes (one to three mm) and simple fractures of different angles (5°–60°) were compared for several levels of plate stroke (0.05–0.60 mm) in three separate healing stages: 1. Initial connective tissue to fibro cartilage, 2. Fibro cartilage to soft callus, and 3. Soft callus to hard callus. All statistical tests were executed with IBM SPSS Statistics version 29 (IBM SPSS Statistics, Armonk, NY, United States). Significance was defined as *p* < 0.05. Normality tests were conducted using the Kolmogorov-Smirnov and Shapiro-Wilk tests. Multiple linear regression analyses were performed with forced entry for each of the named outcome measures as the dependent variable. Forced entry was chosen, as the number of independent variables is low and all variables have an explainable influence (Kucuk et al., 2016). The relationships of each of these parameters with the fracture gap size or angle, and the stroke of the plate as independent variables were explored. Two models were computed for each parameter, one for fracture gap size, and one for fracture angle. The reason why they could not be entered in the same model is that these are two separate data sets. The adjusted *R* ^2^ for each dependent variable delivered by the multiple linear regression analysis indicates the percentage of the variability explained by the independent variables.

**TABLE 1:** Outcome measures in the fracture gap computed with finite element simulations.

Outcome measure	Description	Unit
J2	The second invariant of the deviatoric strain tensor, usually labelled J2, was identified as key quantity to describe the influence of strain on fracture healing (Garcia et al., 2003; Doblaré et al., 2004). It reflects the interplay of local volume change and local shape distortion. This was experimentally underpinned in the work of Bishop and colleagues (Bishop et al., 2003)	-
Strain energy density (SED)	In general, the strain energy density is a measure of how much energy a material can store and release when it is deformed. In the modelling of fracture healing and even more for bone remodelling, SED is often interpreted as a form of stimulus that causes adaptations (Weinans et al., 1992)	N/mm2
Hydrostatic strain (HS)	Hydrostatic strain is the average of the three normal strains of any strain tensor and contributes to the volume change (Ghiasi et al., 2017)	-
Octahedral shear strain (OSS)	The maximum shear strain in any plane for a 3D state of strain. OSS, derived from the deviatoric part, contributes to shape distortion (Ghiasi et al., 2017)	-
Percentage of fracture gap in the ‘perfect healing window’	Percentage of elements in the fracture gap with a deformation state corresponding to the mechanical stimulus identified as promoting healing (Claes and Heigele, 1999; Shefelbine et al., 2005), as used in (Braun et al., 2021; Orth et al., 2023)	Percent

## 3 Results

All data were normally distributed. All multiple linear regression models were significant, which means they could be used.

### 3.1 Perfect healing window

**FIGURE 2:**
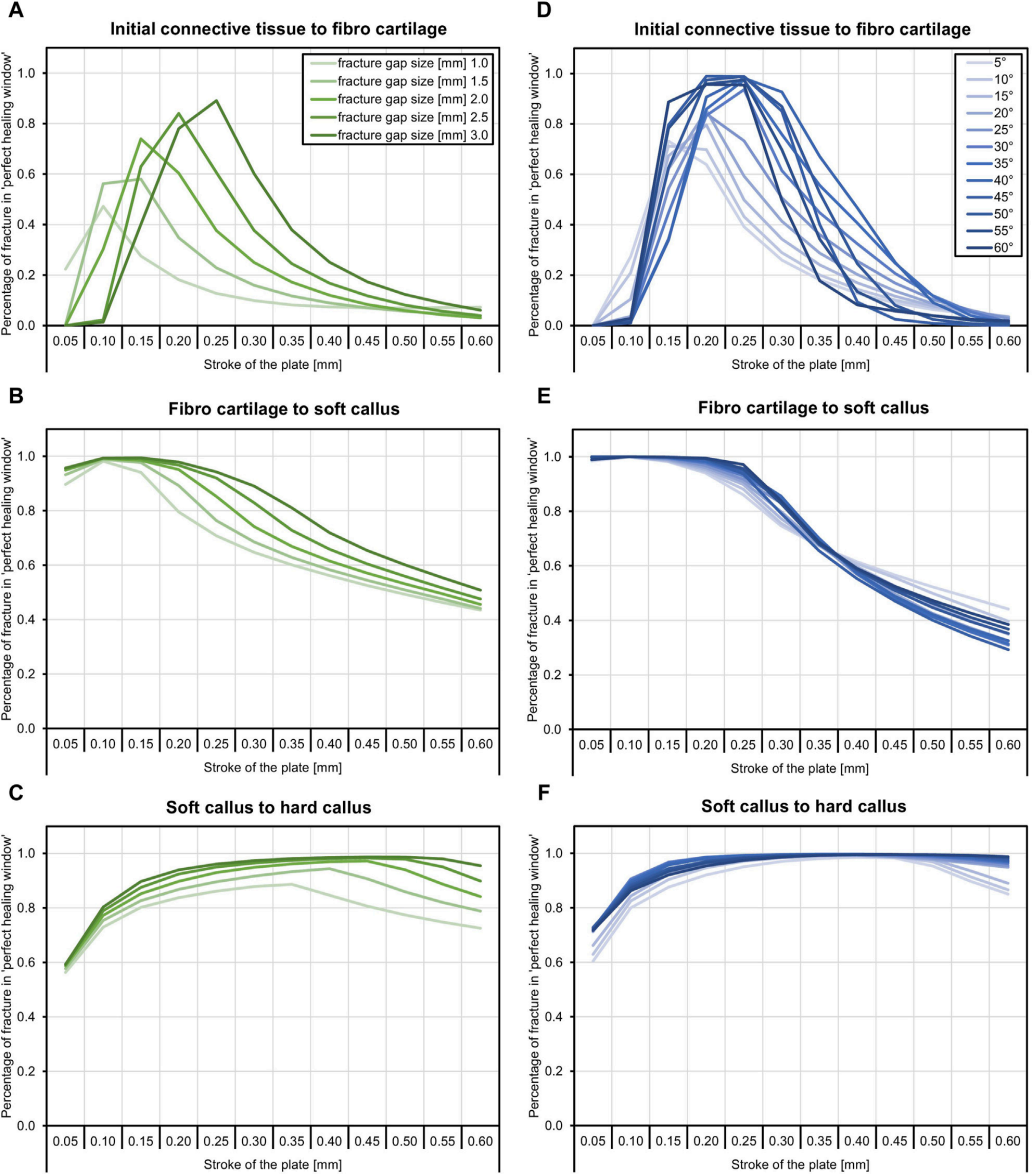
The proportion of the fracture gap in the perfect healing window depends on the fracture gap size, fracture angle, the axial stroke of the active implant, and the healing stage. The plate stroke and angle leading to the highest proportion of the fracture gap in the perfect healing window depends on the fracture gap size or fracture angle and healing stage. **(A)** In the early fracture healing phase, results indicate that, depending on the fracture gap size, an axial plate stroke of around 0.1 mm–0.25 mm is ideal (see Table 2). This is around 10% of the fracture gap size. **(B)** In the fibro cartilage to soft callus-phase, the ideal stroke is between 0.1 mm and 0.15 mm for all fracture gap sizes. **(C)** In the late healing phase, the ideal stroke increases with the fracture gap size and is between 0.35 mm and 0.45 mm. **(D)** With fracture obliquity, in the initial healing phase, the ideal stroke increases with pronounced maxima between 0.15 mm and 0.25 mm. **(E)** The second healing phase shows only small differences between fracture angles and very low ideal stroke values of 0.05 mm and 0.1 mm, while ideal values increase to 0.3 mm–0.45 mm in the last healing phase **(F)**.

**FIGURE 3:**
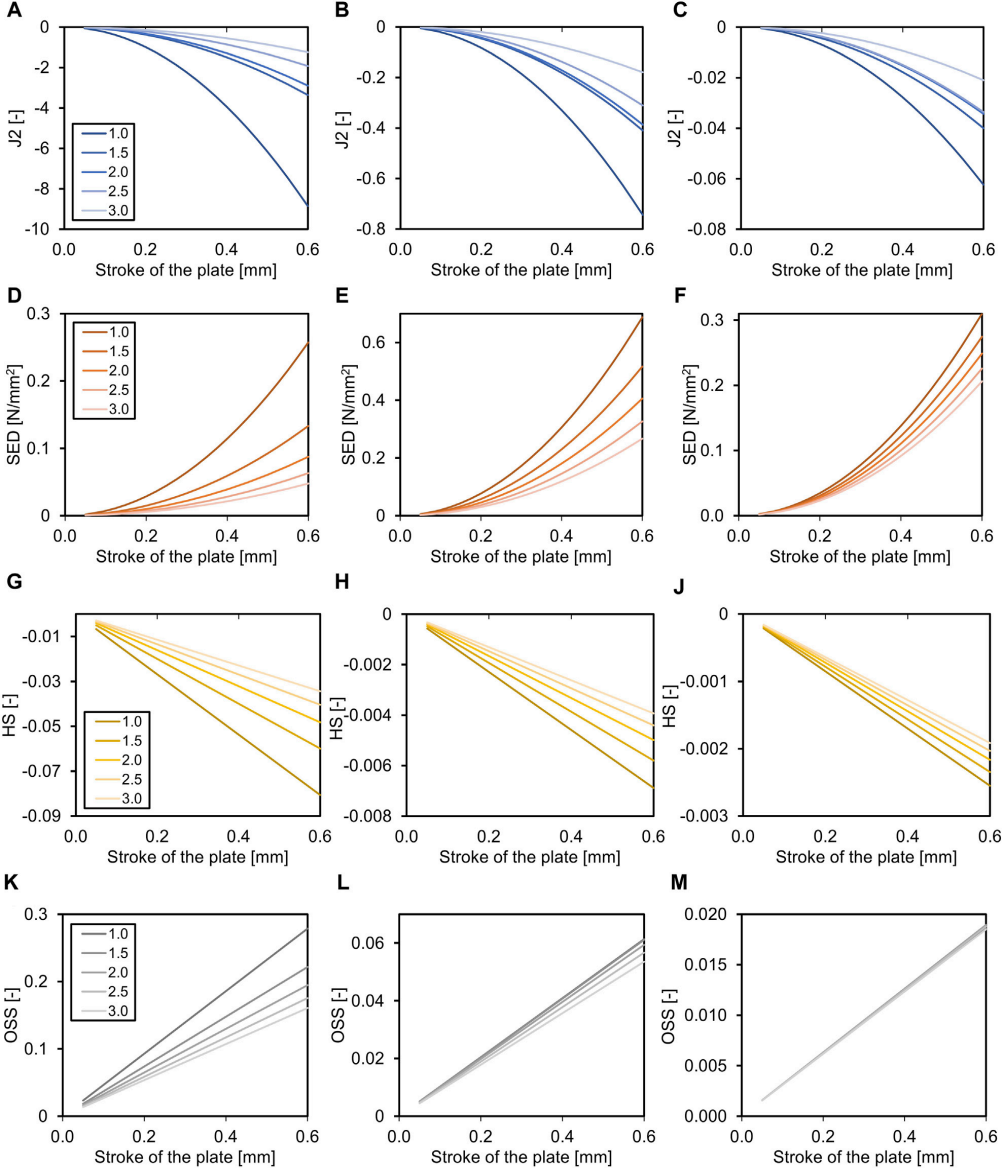
Effects of the fracture gap size (for transverse fracture, legend shows mm fracture gap) on the required axial stroke in the plate. Relation of the mean J2 **(A–C)**, the mean strain energy density (SED, **D–F**), the hydrostatic strain (HS, **G–J**), and the octahedral shear strain (OSS, **K–M**) with the axial stroke of the plate and the fracture gap size (distance between bone ends). While the relation is non-linear for J2 and SED, it is linear for HS and OSS. Data are shown for the three analyzed phases of fracture healing separate, initial connective tissue to fibro cartilage **(A,D,G,K)**, fibro cartilage to soft callus **(B, E, H, L)**, and soft callus to hard callus **(C, F, J, M)**. Note the different dimensions on the Y-axes throughout healing.

**FIGURE 4:**
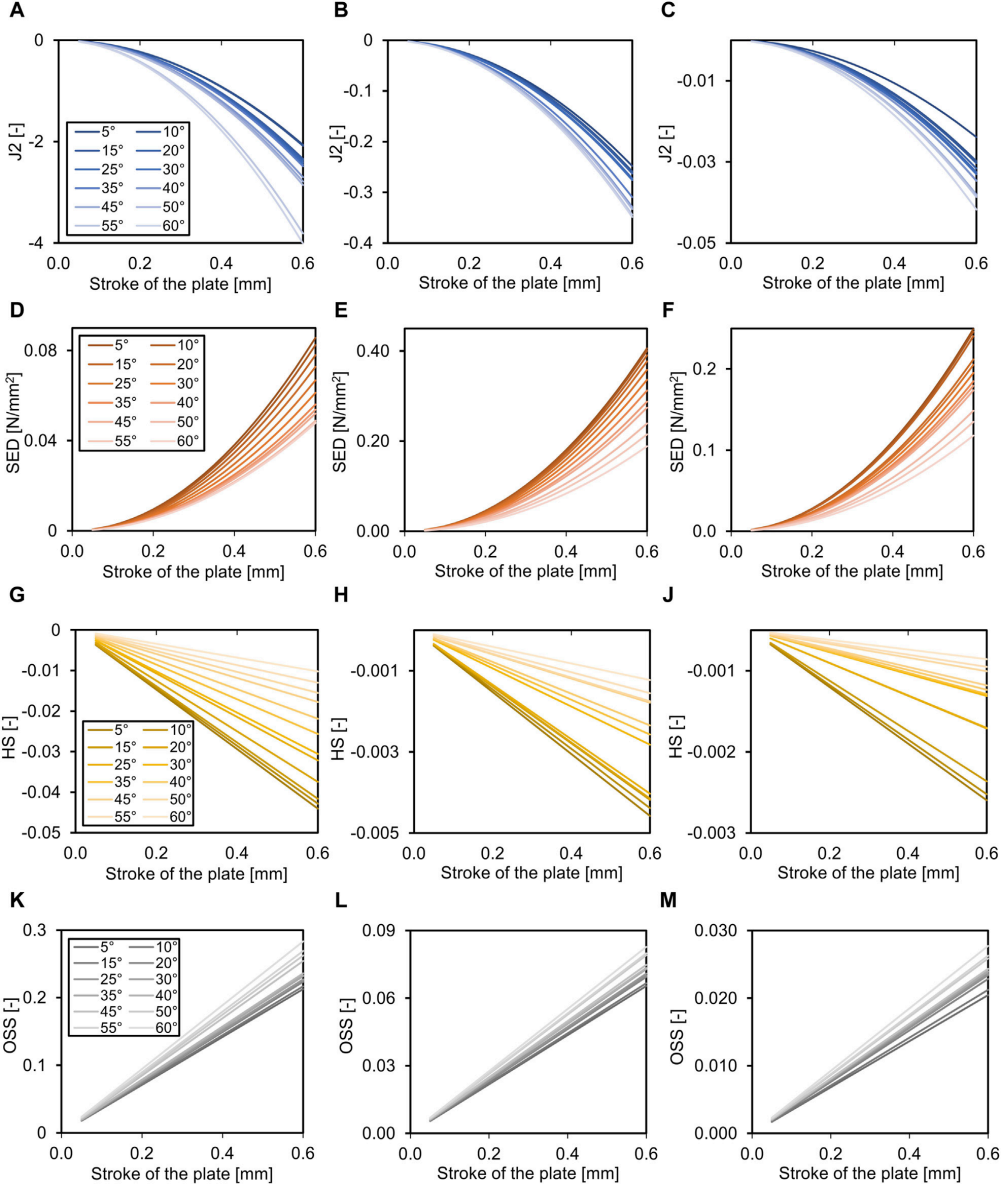
Effects of the fracture angle (for oblique fracture, legend shows degrees fracture angle) on the required axial stroke in the plate. Relation of the mean J2 **(A–C)**, the mean strain energy density (SED, **D–F**), the hydrostatic strain (HS, **G–J**), and the octahedral shear strain (OSS, **K–M**) with the axial stroke of the plate and the fracture angle. While the relation is non-linear for J2 and SED, it is linear for HS and OSS. Data are shown for the three analyzed phases of fracture healing separate, initial connective tissue to fibro cartilage **(A,D,G,K)**, fibro cartilage to soft callus **(B, E, H, L)**, and soft callus to hard callus **(C, F, J, M)**. Note the different dimensions on the Y-axes throughout healing.

Figure 2 shows the proportion of the fracture gap in the perfect healing window in relation to the fracture angle and the axial stroke performed by the implant. Based on the perfect healing window-data, the best axial plate-movement values for each fracture size (distance between bone ends) and healing phase are shown in Table 2. The ideal axial plate-movement values for different angles in oblique fractures are shown in Table 3. Findings indicate that an active fracture plate should deliver an axial stroke in the range of 0.1–0.45 mm to be able to cover the tested fracture types (Tables 2; 3; Figure 2 to 4). In addition, the highest adjusted R^2^-values that indicate the percentage of the variability explained by fracture gap size and angle were found in the second healing phase (Tables 4; 5).

**TABLE 2:** Axial plate-movement values that delivered the highest percentage of the fracture gap in the ‘perfect healing window’ in mm for each fracture size (distance between bone ends) and healing phase, based on the perfect healing window-data. Results shown are for transverse tibial shaft fractures. See Figure 2 for visualization.

Healing phase	Fracture gap size [mm]					Range
	1.0	1.5	2.0	2.5	3.0	
Initial connective tissue to fibro cartilage	0.10	0.15	0.15	0.20	0.25	0.10–0.25
Fibro cartilage to soft callus	0.10	0.10	0.10	0.10	0.15	0.10–0.15
Soft callus to hard callus	0.35	0.40	0.45	0.45	0.45	0.35–0.45

**TABLE 3:** Axial plate-movement values that delivered the highest percentage of the fracture gap in the ‘perfect healing window’ in mm for each fracture angle (degree the fracture is tilted) and healing phase, based on the perfect healing window-data. Data shown are for oblique tibial shaft fractures with a 2-mm fracture gap size. See Figure 2 for visualization.

Healing phase	Fracture angle [degrees]												Range
	5°	10°	15°	20°	25°	30°	35°	40°	45°	50°	55°	60°	
Initial connective tissue to fibro cartilage	0.15	0.15	0.20	0.20	0.20	0.25	0.25	0.25	0.20	0.25	0.25	0.25	0.15–0.25
Fibro cartilage to soft callus	0.10	0.10	0.10	0.05	0.10	0.10	0.10	0.10	0.05	0.10	0.10	0.10	0.05–0.10
Soft callus to hard callus	0.40	0.40	0.40	0.30	0.35	0.35	0.40	0.40	0.45	0.45	0.45	0.45	0.30–0.45

**TABLE 4:** Findings from the multiple linear regression analysis for fracture gap size. Adjusted R^2^ values are shown for each dependent variable, and indicate the percentage of the variability explained by the independent variables. As an example, 59.4% of the variability of J2 in the first healing phase is explained by the fracture gap size and the stroke of the plate. *p*-values, non-standardized and standardized (Beta) coefficients of the computed parameters are listed for each parameter and healing phase, if significant. Healing phases: 1 = Initial connective tissue to fibro cartilage, 2 = Fibro cartilage to soft callus, 3 = Soft callus to hard callus.

Parameter	Healing phase	Adjusted R^2^	Fracture gap size	Stroke of plate
J2	1	0.594	<0.001, 0.654, 0.443	<0.001, −6.601, −0.641
	2	0.751	<0.001, 0.048, 0.361	<0.001, −0.734, −0.793
	3	0.817	<0.001, 0.004, 0.307	<0.001, −0.069, −0.854
SED	1	0.680	<0.001, −0.020, −0.460	<0.001, 0.213, 0.692
	2	0.848	<0.001, −0.043, −0.330	<0.001, 0.795, 0.863
	3	0.925	<0.001, −0.011, −0.157	<0.001, 0.457, 0.950
HS	1	0.893	<0.001, 0.007, 0.449	<0.001, −0.088, −0.834
	2	0.952	<0.001, 0.000, 0.329	<0.001, −0.009, −0.919
	3	0.986	<0.001, 0.000, 0.180	<0.001, −0.004, −0.977
OSS	1	0.942	<0.001, −0.017, −0.316	<0.001, 0.344, 0.919
	2	0.996	<0.001, −0.001, −0.087	<0.001, 0.097, 0.994
	3	1.000	0.450	<0.001, 0.031, 1.000
Percent of fracture gap in the ‘perfect healing window’	1	0.206	0.054	<0.001, −0.578, −0.425
	2	0.933	<0.001, 0.036, 0.226	<0.001, −1.055, −0.940
	3	0.368	<0.001, 0.037, 0.397	<0.001, 0.314, 0.481

**TABLE 5:** Findings from the multiple linear regression analysis for fracture angle. Adjusted R^2^ values are shown for each dependent variable, and indicate the percentage of the variability explained by the independent variables. As an example, 89.6% of the variability of J2 in the first healing phase is explained by the fracture angle and the stroke of the plate. *p*-values, non-standardized and standardized (Beta) coefficients of the computed parameters are listed for each parameter and healing phase, if significant. Healing phases: 1 = Initial connective tissue to fibro cartilage, 2 = Fibro cartilage to soft callus, 3 = Soft callus to hard callus.

Parameter	Healing phase	Adjusted R^2^	Fracture angle	Stroke of plate
J2	1	0.896	<0.001, −0.011, −0.217	<0.001, −4.871, −0.922
	2	0.933	<0.001, −0.001, −0.137	<0.001, −0.538, −0.957
	3	0.929	<0.001, −0.000, −0.152	<0.001, −0.061, −0.952
SED	1	0.908	<0.001, 0.000, −0.231	<0.001, 0.113, 0.925
	2	0.905	<0.001, −0.002, −0.249	<0.001, 0.569, 0.919
	3	0.908	<0.001, −0.001, −0.238	<0.001, 0.344, 0.924
HS	1	0.897	<0.001, 0.000, 0.585	<0.001, −0.046, −0.746
	2	0.889	<0.001, 0.000, 0.563	<0.001, −0.005, −0.757
	3	0.824	<0.001, 0.000, 0.644	<0.001, −0.002, −0.641
OSS	1	0.990	<0.001, 0.001, 0.157	<0.001, 0.399, 0.982
	2	0.994	<0.001, 0.000, 0.126	<0.001, 0.122, 0.989
	3	0.992	<0.001, 0.000, 0.143	<0.001, 0.040, 0.986
Percent of fracture gap in the ‘perfect healing window’	1	0.120	0.165	<0.001, −0.664, −0.346
	2	0.938	0.677	<0.001, −1.393, −0.969
	3	0.344	0.026, 0.001, 0.153	<0.001, 0.282, 0.575

### 3.2 Fracture gap size

The fracture gap size affected all parameters in all healing phases (all *p* < 0.001), except for octahedral shear strain in the third healing phase, and percent of fracture gap in the ‘perfect healing window’ in the first healing phase (Table 4). Figure 3 shows the relation of the outcome measures with the axial stroke of the plate and the fracture gap size (thickness of the fracture gap) for each healing phase. The smaller the fracture gap, the larger was the effect on the outcome measures. Results of the multiple linear regression analysis are shown in Table 4. The highest adjusted R^2^-values that indicate the percentage of the variability of J2, SED, HS, and OSS explained by fracture gap size and plate stroke were found in the last healing stage (Table 4). However, this was different for the percentage of the fracture gap in the perfect healing window, where *R* ^2^ for fracture gap size and plate stroke was highest in the second healing phase.

### 3.3 Fracture angle

The fracture angle affected all parameters in all healing phases (*p* < 0.001), except for the percentage of the fracture gap in the ‘perfect healing window’, that did not correlate with the fracture angle in the first two healing phases (Table 5). Figure 4 shows the relation of the outcome measures with the axial stroke of the plate and the fracture angle for each healing phase. For J2 and octahedral shear strain, a greater fracture angle led to a greater effect, while for SED and HS, the opposite was found (Figure 4). The adjusted R^2^-values for fracture angle and plate stroke were equally distributed throughout healing phases for J2, SED, HS, and OSS, while the adjusted *R* ^2^ for the percentage of the fracture in the perfect healing window was highest in the second healing phase (Table 5).

### 3.4 Healing phases

The ideal axial stroke in the plate was 0.10–0.25 mm for the first, 0.10 mm for the second, and 0.35–0.45 mm for the third healing phase when considering the finding of both analyses combined (Tables 2, 3). For each of the computed parameters, Figures 2–4 show differences in the scales on the *Y*-axes, which reflects changes in the local mechanical properties with fracture healing. The influence of fracture gap size and plate stroke on the adjusted *R* ^2^ for J2, SED, HS, and OSS was greatest in the last healing phase, while fracture angle and plate stroke did not show a peak at any healing phase (Tables 4, 5). For the percentage of the fracture in the perfect healing window, adjusted *R* ^2^ was greatest in the second healing phase for both fracture gap size and fracture angle.

## 4 Discussion

The present study showed that an active fracture plate should be able to deliver an axial stroke in the range of 0.10–0.45 mm to cover the tested fracture types. Different optimal stroke values are required for each healing phase, namely, 0.10–0.25 mm for the first, 0.10 mm for the second, and 0.35–0.45 mm for the third healing phase, depending on the fracture gap size (distance between bone ends), but less dependent of the fracture angle. The computed strain values and the strain energy density correlated with the fracture gap size and angle. In detail, analyses revealed that both fracture gap size and fracture angle had the greatest contribution to the variability of the percentage of the fracture gap in the perfect healing window during the second healing phase. For the other biomechanical parameters, the fracture gap size showed the greatest contribution in the third fracture healing phase, and the influence of fracture angle was independent of the healing phase. Based on the presented findings, the null-hypothesis that the resulting strain in the fracture gap of a simple tibial shaft fracture does not vary with the amount of axial stroke in the plate, the fracture gap size, and the fracture angle, was rejected.

It has long been known that the biomechanical requirements for rapid fracture healing vary with the stage of healing (Wolf et al., 1981). The dimensions of movement in the present study are in the range of experimental findings from Kenwright & Goodship (Kenwright and Goodship, 1989), who showed that 0.5 mm of axial micromovement was better than 2 mm. The present study would recommend 0.25 mm for the first, 0.15 mm for the second, and 0.45 mm for the third phase for a 3-mm transverse fracture gap. This is the first time that the required movement has been calculated to such detail. The present paper therefore opens up and demonstrates the methodology and which data such calculations can provide. It also showcases the non-linearity and complexities of determining the ideal stroke of an active plate. While the present simulations only covered the fracture angle and fracture gap type, this could in the future be expanded to more complex fracture types, such as wedge fractures, where the rotation of the wedge will likely play a major role. An increasing fracture gap size also leads to a longer duration of the healing process, which is another factor that may be addressed in future simulations (Claes et al., 1998). Comminuted fractures (several fracture gaps and bone fragments) will be able to tolerate relatively greater motion since the strain is applied over a larger surface area of fracture fragments (Glatt et al., 2017). Shear movement is known to have negative effects on fracture healing (Augat et al., 2003), but while this was considered in the present simulations, results still indicated that the fracture angle did not matter as much as the fracture gap size. The fracture angle could have affected other biomechanical variables, such as shear stress and contact pressure, but at least shear stress does not seem to have influenced the findings to a larger degree. The present results also indicate that the influence of the fracture gap size and angle on the variability in several biomechanical parameters in the fracture gap varies throughout healing. This was previously known and recently increasingly considered, but not yet to this extent (Barcik and Epari, 2021).

The tibial load during movement and gait is influenced by anthropometric parameters such as age, body height, body weight, and hand grip strength, and has an influence on the fracture gap mechanics (Wolff et al., 2023; Wickert et al., 2024). Moreover, muscle contractions (Yang et al., 2015) and behavioral aspects influence the load, such as whether a patient adheres to the partial weight bearing instructions (Ganse et al., 2016), and by how much and how the person moves in daily life (Warmerdam et al., 2023; Warmerdam et al., 2024). In intact bone, the *in-vivo* deformation of the proximal in relation to the distal tibia in terms of bending and torsion during walking and running in humans amounts to up to 0.38°–0.90° of medial bending, 0.15°–1.30° of posterior bending, and 0.67°–1.66° of external torsion (Yang et al., 2014). It is therefore crucial to provide sufficient stiffness by the active implant when the patient walks to shield the fracture gap from excess movement and strain. In addition, the fracture callus stiffens throughout healing, which needs to be considered (Seide et al., 2012; Barcik and Epari, 2021). Therefore, fracture plates with adjustable stiffness have been suggested and introduced (Epari et al., 2021; Ganse et al., 2022). It is, however, due to play unlikely that such an implant will be able to stop all movement in the fracture gap when the patient walks. Thus, it seems of interest to measure the actual movement in the fracture, i.e., indirectly by measuring bending and strain in the fracture plate (Hente et al., 2003; Wickert et al., 2024). When combined with electronics, the patient could then be warned of the excess load, and in a control loop, the implant stiffness could, at least to a certain degree, be adapted. Such warning systems have already been studied using instrumented insoles, but to date, the authors are not aware of any fracture implant with this capability (Abbott et al., 2019; Zhang et al., 2023). Moreover, the axial micromovement would only be needed, if these loads did not already exceed the strain and stimulation time considered optimal for healing. Fracture healing seems to underlie a circadian rhythm and requires daily stimulation, as well as sufficient recovery times of unloading (Barcik and Epari, 2021; Windolf et al., 2022). It also requires stimulation immediately after the injury to gain the best healing results (Barcik et al., 2023). Therefore, it seems desirable to combine sensing and acting capabilities in a smart implant for fracture healing.

The motivation for the present study was the need to know the amount of axial stroke an active implant would need to provide, as the authors required this information for their implant design and construction process. For computer simulations, the suitability and validity of simulation results are often questioned. In this case, however, test stand or animal experiments were not an option, as they would not have been able to provide the needed information that is true for human patients *in-vivo*. Among computer simulation methods, finite element simulations and rigid body assumptions are currently the gold standard for implant development (Mühling et al., 2021). When selecting the outcome measures, the authors sought to cover a variety of aspects that are known to matter in fracture healing. Future *in silico* studies may consider further implant-related factors, such as stress shielding, or soft tissue mechanics including the pull of muscles. In addition, instead of simulating separate healing phases, with more computing power, continuous simulations over the entire healing period may be beneficial. These could include loading data from real patients.

Once an active fracture plate has made it to the market, large-scale randomized clinical trials should be conducted systematically to determine the ideal stimulation setting (frequency, stroke, time) for each fracture type and fracture size. Apart from the parameters considered in the present study, findings may also differ with, i.e., patient age and sex (Tang et al., 2022). Since diseases such as diabetes and osteoporosis are known to delay fracture healing (Nikolaou et al., 2009), it would be desirable to adjust the stimulation pattern individually, and to find ways to assess the fracture healing phases based on measurements. Such studies would require a multi-centre setup and a large number of patients to accommodate for the many different options when varying the settings. The findings of the present study then may serve as a first indication of what to look at. This study also showed that the demand for the amount of axial stroke in the implant and also the contribution of the movement to the variability in biomechanical parameters in the fracture gap changes throughout healing. This should be taken into account when planning the interventions for such studies.

Limitations of this study were as follows: The assumption of three healing stages instead of a continuous, longitudinal simulation is certainly a simplification. However, since the present study already generated 768 simulation results with approximately 1.47 TB of data volume and required a long computing time, longitudinal simulations were not realistic at this level of detail with the simulation methods and hardware used. The concept of the ‘perfect healing window’ invented by Lutz Claes (Claes et al., 1998; Claes and Heigele, 1999) was chosen for the present study, but it is also a simplification of a more complex process. Only one bone of one patient was used to generate the model in this study, while running the simulations on a diverse set of many bones may have delivered a better view on the applicability to the overall population, making results more generalizable. The general rules described in the present findings are likely to be transferrable, while details in the actual stroke values may differ and show variation among patients, fractures and implants. The presented findings are likely only valid for the tibial diaphysis and may differ considerably for other bones, and the metaphyseal and epiphyseal regions. Limitations are also generally present in *in silico* studies that never fully represent the complexities of the real world, and therefore, experiments in animals and humans are required to confirm the findings. One limitation of our *in silico* study is the choice of an isotropic material model instead of an anisotropic model for the bone. However, this is a common simplification in literature due to computing time and the very few published works on anisotropic models (Knowles et al., 2016). A second simplification made in our *in silico* study is the rigid connection between the bone and screws instead of defining contact conditions. This approach is justified because it significantly reduces the computational complexity and time required for the simulations.

Another aspect of *in silico* studies based on FEA results is the question of validation. This is particularly challenging in our case because, on the one hand, the active implant exists only virtually, making experimental verification and validation impossible. On the other hand, the study relies on the bone of a donor, which was scanned via CT and then used for experiments in a different study, so the bone was no longer available at the time of this *in silico* study. Despite these challenges, an initial study (Wickert et al., 2024) has been published that compares the simulation workflow used here with experiments conducted on a testing device. This device applies the forces that occur during a forward step on a bone-implant system, providing an initial validation of the simulation process.

## 5 Conclusion

Based on finite element simulations, recommendations for the axial stroke of an active fracture plate were given for each healing phase, fracture size and angle. Results showed that an active fracture plate should deliver an axial stroke in the range of 0.10–0.45 mm. Different optimal stroke values were found for each healing phase, namely, 0.10–0.25 mm for the first, 0.10 mm for the second, and 0.35–0.45 mm for the third healing phase, depending on the fracture gap size and less on the fracture angle. The influence of the fracture gap size and angle on the variability in several outcome measures in the fracture gap was shown to vary throughout healing. The present findings may be useful for the development of smart fracture implants and to design clinical studies that will be needed to confirm the optimal settings for clinical use.

## Data Availability

The datasets generated and analyzed for this study are available from the authors upon reasonable request. The raw data supporting the conclusions of this article will be made available by the authors, without undue reservation.
